# Supplementary material for: Association of Maternal Plasma Manganese with the Risk of Spontaneous Preterm Birth: A Nested Case–Control Study Based on the Beijing Birth Cohort Study (BBCS) in China
Source: Nutrients. 2023 Mar 15;15(6):1413. doi: 10.3390/nu15061413 (PMC10053178; doi:10.3390/nu15061413)
Supplement: Supplementary file 1 [file nutrients-15-01413-s001.zip › nutrients-2233836-supplementary.pdf]

Table S1 Association of inflammatory factors in Blood routine test with SPB

Factor	OR	95%CI		P
		LOW	High	
First Trimester				
WBC	1.166	1.059	1.285	0.002
GR%	1.033	1.002	1.064	0.036
NE	1.028	0.975	1.084	0.304
EO	1.54	0.3	7.706	0.605
EO%	0.998	0.833	1.197	0.986
MONO	3.783	0.829	17.259	0.086
MONO%	0.876	0.74	1.036	0.121
PLT	1.007	1.003	1.01	＜0.001
PCT	244.516	6.317	9464.438	0.003
Third Trimester				
WBC	1.117	1.022	1.221	0.015
GR%	1.006	0.974	1.04	0.717
NE	1.121	1.014	1.239	0.025
EO	3.614	0.274	47.635	0.329
EO%	1.055	0.912	1.222	0.471
MONO	2.045	0.706	5.923	0.187
MONO%	0.977	0.863	1.107	0.719
PLT	1.006	1.002	1.009	0.004
PCT	294.544	6.596	13151.92	0.003

Unconditional logistic regression was used for analysis. SPB: spontaneous preterm birth; OR: odds ratio; CI: confidence interval. WBC: white blood cell count; GR%: Granulocyte Ratio; NE: neutrophil; EO: Eosinophil; EO%: Eosinophil Ratio; MONO: monocytes; MONO%: monocytes Ratio; PLT: platelet count; PCT: platelet crit.

Table S2. Median Mn concentrations of women who had SPB and term birth stratified by parity

	Median (IQR) (ng/ml)			P
	Total	SPB	TB	
Nulliparous				
n(%)	343 (70.3)	157 (64.3)	186 (76.2)	
First Trimester	0.80(0.59)	0.82(0.62)	0.76(0.60)	0.257
Third Trimester	1.23(0.70)	1.27(0.71)	1.21(0.64)	0.127
Multiparous				
n(%)	145 (29.7)	87 (35.7)	58 (23.8)	
First Trimester	0.86(0.79)	0.84(0.83)	0.94(0.67)	0.644
Third Trimester	1.23(0.66)	1.27(0.66)	1.18(0.62)	0.534

Mn: manganese; SPB: spontaneous preterm birth; TB: term birth; IQR: interquartile range; Bonferroni correction were used here and the adjust significant P value was 0.05/2.

Table S3. Association of maternal plasma Mn level with SPB stratified by parity

Tertile concentration of Mn (ng/ml)	Total n(%)	cases n(%)	controls n(%)	crude OR (95%CI)	P	Adjusted OR[a] (95%CI)	P	Adjusted OR[b] (95%CI)	P	Adjusted OR[c] (95%CI)	P
Nulliparous	343										
First Trimester											
＜0.609	115(33.5)	48(30.6)	67(36)	1		1		1		1	
0.609-1.012	121(35.3)	55(35)	66(35.5)	1.16(0.70-1.95)	0.565	1.15(0.68-1.95)	0.608	1.10(0.64-1.86)	0.738	1.14(0.66-1.97)	0.640
＞1.012	107(31.2)	54(34.4)	53(28.5)	1.42(0.84-2.42)	0.193	1.51(0.87-2.59)	0.140	1.37(0.79-2.37)	0.257	1.47(0.84-2.57)	0.181
P trend				0.193		0.141		0.26		0.182	
Third Trimester											
＜1.061	118(34.5)	49(31.2)	69(37.3)	1		1		1		1	
1.061-1.470	110(32.2)	49(31.2)	61(33)	1.13(0.67-1.91)	0.645	1.12(0.65-1.93)	0.677	1.13(0.66-1.92)	0.668	1.08(0.62-1.89)	0.777
＞1.470	114(33.3)	59(37.6)	55(29.7)	1.51(0.90-2.54)	0.119	1.46(0.86-2.49)	0.161	1.62(0.94-2.77)	0.08	1.54(0.89-2.67)	0.121
P trend				0.12		0.161		0.081		0.121	
Multiparous	145										
First Trimester											
＜0.609	48(33.1)	29(33.3)	19(32.8)	1		1		1		1	
0.609-1.012	42(29)	27(31)	15(25.9)	1.18(0.50-2.78)	0.706	1.11(0.42-2.92)	0.833	1.18(0.49-2.85)	0.717	1.17(0.44-3.13)	0.749
＞1.012	55(37.9)	31(35.6)	24(41.4)	0.85(0.39-1.86)	0.677	0.74(0.29-1.87)	0.559	0.91(0.40-2.07)	0.830	0.86(0.33-2.26)	0.805
P trend				0.658		0.512		0.816		0.762	
Third Trimester											
＜1.061	45(31)	26(29.9)	19(32.8)	1		1		1		1	
1.061-1.470	52(35.9)	28(32.2)	24(41.4)	0.85(0.38-1.91)	0.698	1.24(0.48-3.20)	0.658	0.89(0.39-2.06)	0.790	1.24(0.46-3.35)	0.663
＞1.470	48(33.1)	33(37.9)	15(25.9)	1.61(0.69-3.76)	0.274	2.35(0.86-6.45)	0.095	1.64(0.68-3.95)	0.267	2.29(0.82-6.36)	0.107
P trend				0.272		0.092		0.268		0.107	

Mn: manganese; SPB: spontaneous preterm birth; cases contain women with SPB; Controls contain women with term birth; OR: odds ratio; CI: confidence interval.

a: Unconditional logistic regression with adjustment for age, BMI, education, economy, nationality, parity, gravida, fetal gender, and samplingtime.

b: Unconditional logistic regression with adjustment for inflammatory factors related to SPB in blood routine test. OR was adjusted by white blood cell count (WBC), platelet count (PLT), Granulocyte Ratio (GR), and platelet crit (PCT) in the first trimester; and adjusted by WBC, PLT, PCT, andneutrophil (NE) in the third trimester.

c: Unconditional logistic regression with adjustment for age, BMI, education, economy, nationality, parity, gravida, fetal gender blood week, and inflammatory factors related to SPB in blood routine tests.

Bonferroni correction were used here and the adjust significant P value was 0.05/2.
